# Exploring Potent Fungal Isolates from Sanitary Landfill Soil for In Vitro Degradation of Dibutyl Phthalate

**DOI:** 10.3390/jof9010125

**Published:** 2023-01-16

**Authors:** Shriniketan Puranik, Livleen Shukla, Aditi Kundu, Deeba Kamil, Sangeeta Paul, Govindasamy Venkadasamy, Rajna Salim, Sandeep Kumar Singh, Dharmendra Kumar, Ajay Kumar

**Affiliations:** 1 Division of Microbiology, ICAR-Indian Agricultural Research Institute, New Delhi 110012, India; 2 Division of Agricultural Chemicals, ICAR-Indian Agricultural Research Institute, New Delhi 110012, India; 3 Division of Plant Pathology, ICAR-Indian Agricultural Research Institute, New Delhi 110012, India; 4 Division of Entomology, ICAR-Indian Agricultural Research Institute, New Delhi 110012, India; 5 C.M.B. College, Deorh, Ghoghardiha, Madhubani 847402, India; 6 Centre of Advanced Study in Botany, Banaras Hindu University, Varanasi 221005, India

**Keywords:** *Aspergillus flavus*, bioremediation, esterase, degradation pathway, gas chromatography-mass spectrometry, phthalates

## Abstract

Di-n-butyl phthalate (DBP) is one of the most extensively used plasticizers for providing elasticity to plastics. Being potentially harmful to humans, investigating eco-benign options for its rapid degradation is imperative. Microbe-mediated DBP mineralization is well-recorded, but studies on the pollutant’s fungal catabolism remain scarce. Thus, the present investigation was undertaken to exploit the fungal strains from toxic sanitary landfill soil for the degradation of DBP. The most efficient isolate, SDBP4, identified on a molecular basis as *Aspergillus flavus*, was able to mineralize 99.34% dibutyl phthalate (100 mg L^−1^) within 15 days of incubation. It was found that the high production of esterases by the fungal strain was responsible for the degradation. The strain also exhibited the highest biomass (1615.33 mg L^−1^) and total soluble protein (261.73 µg mL^−1^) production amongst other isolates. The DBP degradation pathway scheme was elucidated with the help of GC-MS-based characterizations that revealed the formation of intermediate metabolites such as benzyl-butyl phthalate (BBP), dimethyl-phthalate (DMP), di-iso-butyl-phthalate (DIBP) and phthalic acid (PA). This is the first report of DBP mineralization assisted with *A. flavus*, using it as a sole carbon source. SDBP4 will be further formulated to develop an eco-benign product for the bioremediation of DBP-contaminated toxic sanitary landfill soils.

## 1. Introduction

Plastics contain a porous polymer backbone with pores filled by a particular class of chemicals called phthalates. Phthalate esters (PAEs) are major additives in plastics manufacturing that constitute around 10–70% of plastic commodities, contributing to their elasticity [1]. Although, Asia-Pacific and Europe have the highest global demands for phthalates and, according to a latest study, the production of PAE accounted for 8 million tons in 2011 globally [2]. Due to a sudden spike in demand for plastic commodities, the phthalic anhydride (a primary component of phthalates) market is estimated to reach USD 5.6 billion at a Compound Annual Growth Rate (CAGR) of 4.2% by 2030 from USD 3.7 billion in 2020, as stated in a survey by Allied market research market outlook [3]. Such bulk use of PAEs has resulted in their omnipresence. Since PAEs are not tightly (chemically) bound to the polymer surface, they leach immediately and contaminate surrounding matrices [4].

Structurally, phthalates have been differentiated into long and short-chain molecules based on the chemical nature of the side chains attached to the phthalic anhydride moiety. Di-butyl phthalate (DBP) is a low molecular weight, majorly used short-chain phthalate. It has diverse applications in cosmetics, children’s toys, automobile wash accessories, explosives, furnishings, adhesives and agrochemicals such as pesticides and fertilizers, gums, inks, plastic films, medical devices, etc. [5,6,7]. It is ubiquitous in the biotic and abiotic natural components of air, water bodies, aquatic animals, sediments, wastewater, soil, plants and humans. A study conducted in Asan Lake in Korea revealed that out of 14 PAEs, DBP was found to be in higher concentrations after di-ethylhexyl phthalate (DEHP) in the surrounding air, water, sediment, and fish of the water body [8]. Later, DBP was reported in high amounts in the urban secondary effluents of China (20.6–103.4 ng L^−1^) [9]. Furthermore, considerable quantities were found in humans, especially in the serum and sweat [10]. DBP can accumulate in humans in numerous ways, including continuous consumption of vegetables grown in contaminated soils. Such soils have been considered a significant concern because they act as a full sink for DBP, which can be transported into plants. Kong et al. [11] concluded that DBP concentrations in soils, films used for mulching and vegetables grown under plastic mulches were found to be 0.42–1.23, 2.3–11.2 and 0.11–2.95 mg kg^−1^, respectively. In addition, various phthalates, including DBP, have also been detected in leachate and municipal landfill soils [12].

A survey of the literature revealed that DBP caused a diverse range of health complications, including endocrine disruption, sterility, birth problems, teratogenic issues, neural problems, obesity, diabetes, asthma, allergies, etc., in humans [13,14]. Due to its notorious nature, several regulatory bodies have imposed strict regulations against DBP utilizations. All the more so, the Consumer Product Safety Improvement Act [15] has restricted the use of DBP to 0.1% in children’s toys. Furthermore, the European Commission has labelled it a ‘reproductive toxicant’, thereby restricting its usage in the cosmetic industry [16]. Similarly, the United States Environmental Protection Agency (USEPA) classifies DBP as a ‘priority pollutant’ [17], highlighting the urgency of interventions for the degradation of DBP in the environment.

It is a fact that the degradation of short-chain phthalates such as DBP is relatively faster and easier in comparison to long-chain phthalates such as DEHP [18]. Though, natural processes of photolysis and hydrolysis are considered to be rapid and efficient means of degradation of DBP [19,20,21]. However, very few attempts were made previously to understand the mechanisms using microbial-assisted interventions for phthalate degradation [22,23,24,25,26,27]. Microorganisms employ a diverse range of enzyme compositions consisting of esterases, dioxygenases, cutinase, etc., and they play a crucial role in degradation [20]. In previous studies, various bacterial genera of *Delftia, Enterobacter*, *Agrobacterium, Variovorax, Micrococcus, Gordonia, Methylobacillus, Rhizobium, Bacillus* and *Rhodococcus* have been reported to degrade phthalates [21,28,29,30,31,32,33,34,35,36,37]. Among fungi, *Polyporus, Fusarium* and *Pleurotus* have been studied previously [38,39,40]. In a study, white rot fungus, *Polyporus brumalis*, was inoculated in shallow stationary culture with DBP (100 µM), which resulted in complete degradation after 18 days [38]. Similarly, Ahuactzin-Pérez et al. [39] tested DBP degradability using *Fusarium culmorum* and found that the fungus broke down 99.30% DBP (500 mg L^−1^) after 9.5 days. In another study, *Pleurotus ostreatus* mineralized >99% DBP (500 mg L^−1^) within 13 days of inoculation [40]. Conversely, these studies included glucose for initial fungal growth along with DBP, which was not used in the present investigation. Assessment of esterases during DBP metabolism was also used in this work.

Although some of the reports available include fungi-mediated DBP mineralization, no reports are available on DBP degradation or its intermediates with the utilization of *Aspergillus flavus.* Thus, in the present study, an efficient fungus, *A. flavus* SDBP4, was identified from the DBP sanitary landfill soil based on its DBP degradation potential. Furthermore, a correlation was established between the characterizations of the intermediates of the biodegradation pathway and the production of fungal esterases. 

## 2. Materials and Methods

### 2.1. Soil Sampling

Contaminated soil samples were collected in sterilized plastic bags from sanitary landfills (28°73′94.9″ N, 77°15′72.8″ E) near Bhalswa Village, New Delhi, India. The collected representative soil samples were pooled to ensure enough homogenization aseptically and stored under refrigerated conditions at 4 °C for future use.

### 2.2. Enrichment and Isolation of DBP Degrading Fungi

The soil sample (50 g) was added to a modified basal salt medium (BSM, 200 mL) containing (g L^−1^): K_2_HPO_4_ (1.0), NaCl (1.0), NH_4_Cl (0.5), MgSO_4_·7H_2_O (0.4), CaCl_2_ (0.3) and FeSO_4_·7H_2_O (0.003) and maintained at pH 6.5 [22]. The medium was supplemented with DBP (50 mg L^−1^) from stock made with equal volumes of DBP and ethanol. Tween 80 (400 mg L^−1^) was added to the medium to improve DBP’s solubility [41,42]. The same medium composition was used for further degradation experiments. Chemicals used in the study were obtained from Sigma-Aldrich (Bangalore, India). An aliquot (10 mL) was inoculated into the fresh medium after 15 days, and the process was repeated thrice. Subsequently, the suspension was serially diluted and spread over sterilized plates containing BSM supplemented with DBP (100 mg L^−1^) and incubated for 7 days. Pure colonies of fungi were isolated, purified, and maintained on potato dextrose agar (PDA) slants.

### 2.3. Morphological Characterization of Isolates

All the isolated fungal strains were morphologically characterized for colony and sporulation characteristics by growing on potato dextrose agar. The best DBP degrading strain was observed under the Carl Zeiss light microscope (40×).

### 2.4. DBP Biodegradation

The DBP degradation experiment was carried out by inoculating approximately 10^6^ spores of each isolate (4 agar plugs of 10 mm diameter in case of non-sporulating isolate, SDBP6) separately into 50 mL BSM liquid media containing 100 mg L^−1^ DBP. The Triplicate setup was incubated at 150 rpm, 30 ± 2 °C in an incubator shaker (MRC Labs, United Kingdom) for 15 days. Periodically, samples were carried out at 3-, 5-, 7-, 10- and 15-day intervals to estimate fungal biomass, total soluble proteins, and esterase production. Degradation (%) of DBP was assessed at the same time interval in triplicate. The degradation (%) of DBP was calculated using the following equation:

$$\%\mathrm{Degradation}\;\left(Y\%\right)=\frac{\left(C_{i}-C_{f}\right)}{C_{i}}\;\times \;100$$

where *C_i_* = initial DBP concentration and *C_f_* = final DBP concentration.

### 2.5. Determination of Fungal Biomass

The direct wet weight method was used to measure fungal biomass with slight modifications [43]. Briefly, the mycelia at different time intervals (3, 5, 7, 10 and 15 days) were filtered through Whatman no. 1 filter paper and dried overnight at 65 °C. After assuring complete drying, the mycelia were weighed using a digital balance (Precisa 310M, Dietikon, Switzerland).

### 2.6. Determination of Proteins and Esterase Production

Total soluble proteins were estimated using the Bradford method [44]. Briefly, filtrate (50 µL) was added into a 96-well microplate, to which fresh Bradford reagent (250 µL) was added. The plates were incubated for 30 min under dark conditions, and respective absorbance was recorded at 595 nm using a microplate reader (BioTek^®^, Winooski, VT, USA).

Esterase production was measured using the standard procedure with *p*-nitrophenyl-acetate as a substrate [31]. Briefly, 1 mL 0.1 M sodium phosphate buffer (pH 7.5) was added to the culture (1 mL) and centrifuged at 10,752× *g* for 15 min. The supernatant was used as an enzyme source for the other reactions. Further, the enzyme (50 µL) was added to a microplate, to which substrate (200 µL, 1 mM) was added along with the sodium phosphate buffer (25 µL, 0.1 M) (pH 6.0). Media without the enzyme was used as blank in this experiment, and the absorbance was recorded at 405 nm using a microplate reader. One enzymatic unit of esterase activity (U) was defined as the amount of enzyme that produced an increase of one unit of absorbance under assay conditions. Both these parameters were assessed for the 3-, 5-, 7-, 10- and 15-day samples in triplicates.

### 2.7. DBP Extraction and Analysis

Residual DBP was extracted from each treatment and kept at different time points with n-hexane (50 mL) in three batches at 28 ± 1 °C overnight using an incubator shaker (MRC Labs, United Kingdom). The extracts were then filtered through Whatman no. 1, combined, and evaporated under reduced pressure using a rotary evaporator (Heidolph, Germany) below 35 °C to obtain the respective residues. The DBP residue in each treatment was dissolved in gas chromatography-mass spectrometry (GC-MS) grade hexane (2 mL) separately and filtered with a 0.22 µm membrane (Millipore, Billerica, MA, USA).

The samples were analyzed using a 5590C GC-MS (Agilent Technologies^®^, Santa Clara, CA, USA) to estimate residual DBP and metabolites characterizations. Intermediates were separated through the Agilent HP-5MS column (30 m × 0.25 mm, film thickness 0.25 µm) and detected using the mass spectrometer. Samples (1 µL, each) were injected through an auto-injector and analyzed while maintaining the split ratio of 10:1. Helium gas (>99.99% purity) was used as carrier gas with a flow rate of 0.75 mL min^−1^ and pressure of 10 psi. A GC-MS temperature ramping was followed, which was initiated at the temperature of 60 °C and increased at the rate of 15 °C min^−1^ to reach 150 °C, then held for 1 min, and then again enhanced at the rate of 10 °C min^−1^ to reach 220 °C with a wait time of 2 min. Then, the temperature was raised at the rate of 5 °C min^−1^ to reach the level of 300 °C. The total run time was 32 min. The MS acquisition parameters were programmed with the ion source temperature of 250 °C, electron ionization of 70 eV, transfer line temperature of 250 °C, full scan mode (50–550 AMU), solvent delay of 3 min and E.M. voltage of 1220 V. Estimation of DBP and its metabolites were carried out using the reference standard, corresponding retention index (RI), matching with NIST mass spectral library and mass fragmentation pattern [45].

In addition to residual estimation, intermediate DBP metabolites were also characterized using GC-MS chromatogram retrieved from different samples. A schematic biodegradation pathway was proposed based on the identified metabolites in the treated samples at interval time points. The chemical structures of all molecules and pathways were constructed using ChemDraw JS 19.0.0.

### 2.8. Molecular Identification of SDBP4

The fungal strain with the highest DBP degradation percentage was identified using a method reported previously [46]. Briefly, the internal transcribed spacers (ITS) region of genomic DNA was amplified with PCR using the universal primers ITS-1 (5′-TCCGTAGGTGAACCTGCGG-3′) and ITS-4 (5′-TCCTCCGCTTATTGATATGC-3′). The PCR was performed using the following amplification cycle: denaturation at 94 °C for 4 min; followed by 35 cycles of 1 min at 94 °C, 1 min at 55 °C, 2 min at 72 °C; and a final extension of 10 min at 72 °C. The PCR product was sequenced by AgriGenome Labs Pvt. Ltd. (Kochi, India). The sequence was subjected to NCBI BLAST_N_, and a phylogenetic tree was developed using MEGA version 11.0 software (Maximum likelihood method).

### 2.9. Statistical Analysis

The data reported are the mean and standard deviation of the treatments in triplicates. The statistical analysis was performed at a 0.05 level of significance using two-way ANOVA and the OPISTAT software [47].

## 3. Results

### 3.1. Isolation of DBP Degrading Fungi

**Figure 1:**
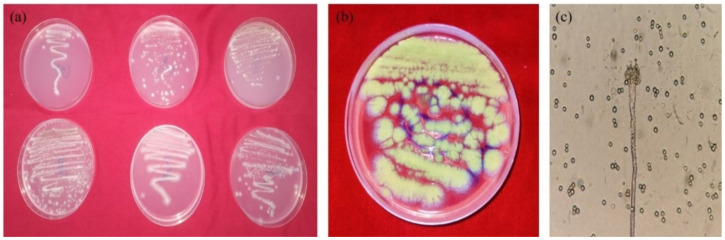
Morphological characteristics of DBP-degrading fungi. (**a**) Six fungal isolates on DBP-containing BSM plates. (**b**) Growth of SDBP4 on a PDA plate. (**c**) SDBP4 under 40× magnification.

In the present study, eleven fungi were isolated from DBP-contaminated sanitary landfill soil after enrichment with DBP as a prime carbon source. Six isolates, namely, SDBP4, SDBP6, SDBP7, SDBP8, SDBP9 and SDBP10 were used for further degradation experiments based on preliminary growth characteristics on the DBP-containing BSM plates. The selected fungi were morphologically different from each other (Figure 1). Their colonial morphology and sporulation are mentioned in Table 1. All the fungi appeared different and were spore producers except SDBP6. While SDBP4 produced white mycelia with light green spores on PDA (Figure 1b,c), SDBP10 formed powdery, dull green spores. Although good fungal proliferation was observed on PDA, their growth in the DBP medium varied.

### 3.2. Biomass, Proteins and Esterase Production

**Figure 2:**
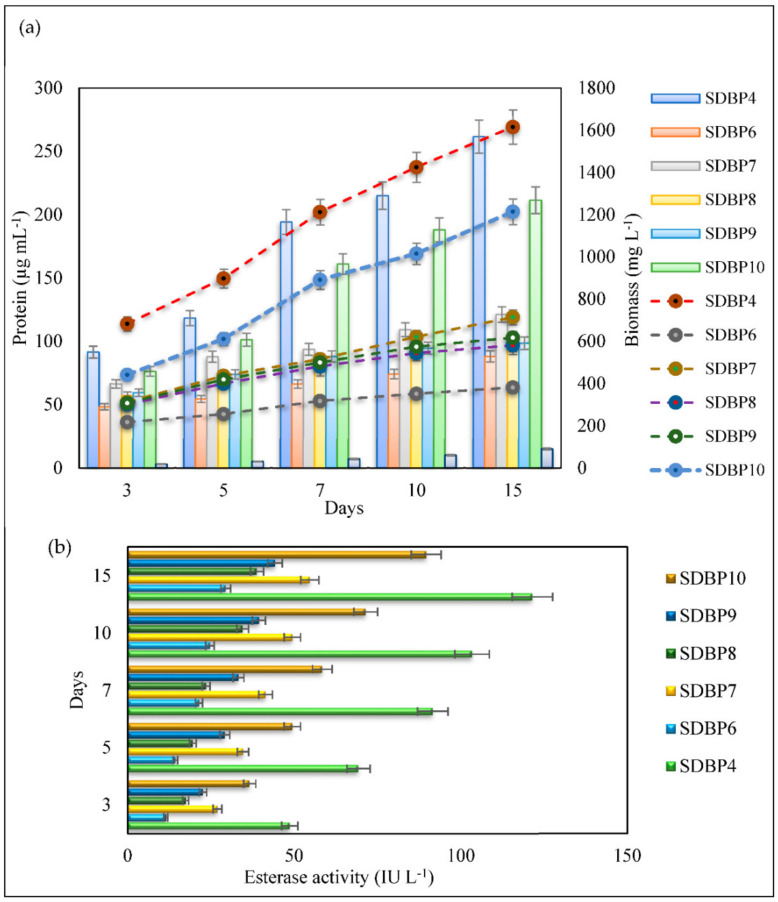
(**a**) Changes in biomass (mg L^−1^) and protein content (µg mL^−1^) in the medium of the different strains during DBP degradation. (**b**) Esterase production (IU L^−1^) by various strains during the degradation of DBP.

The biomass dynamics of isolates during DBP degradation are shown in Figure 2a. With the increase in time, the biomass of the isolates increased, confirming the utilization of DBP as a carbon source. The biomass of all isolates differed significantly and was found to be in the order SDBP4 > SDBP10 > SDBP7 > SDBP9 > SDBP8 > SDBP6 at the end of degradation. Biomass accumulation of SDBP4 showed an increasing trend as the time of degradation increased, achieving the highest growth on the 15th day (1615.33 mg L^−1^). Though SDBP10 exhibited good growth on the 3rd day of degradation (441.33 mg L^−1^), its biomass could only reach 1214.20 mg L^−1^ at the end of the 15th day. SDBP6 showed consistently poor growth during degradation, with final biomass of 381.13 mg L^−1^. A similar trend was observed in the case of total soluble protein content, which differed significantly between the isolates and within the days of degradation. The results revealed that SDBP6 showed the least protein content at the end of the 15th day (88.23 µg mL^−1^). Although SDBP10 produced more protein than SDBP7, SDBP9, SDBP8 and SDBP6 on the 15th day, the amount was significantly less than SDBP4 (Figure 2a). The observations also demonstrated that the interaction between the fungal strains and degradation time factors greatly influenced esterase production. It was evident that as time increased, the isolates synthesized more esterase to degrade DBP. On the 3rd day, esterase production was highest by SDBP4 (48.62 IU L^−1^), followed by SDBP10 (36.61 IU L^−1^), reaching 121.47 IU L^−1^ and 89.63 IU L^−1^, respectively, at the end of the degradation studies. In contrast, SDBP6 had 4.12-fold lesser enzyme production (29.44 IU L^−1^) than SDBP4 on the last day, thus, showing its reduced ability to metabolize DBP (Figure 2b).

### 3.3. Biodegradation of DBP

**Figure 3:**
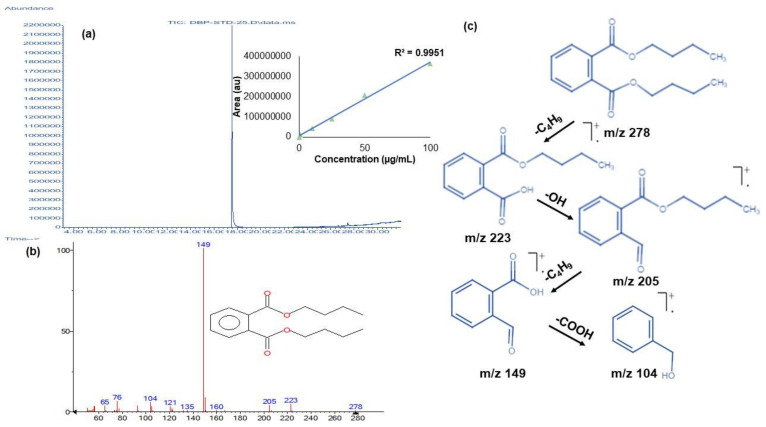
(**a**) Total ion chromatogram (TIC) of DBP as analyzed using GC-MS along with the calibration curve, (**b**) molecular ion peak of DBP along with fragment ions and (**c**) proposed mass fragmentation pattern of DBP. The structures were constructed using ChemDraw JS 19.0.0.

After inoculating all six isolates individually, the characterization of residual DBP was carried out on 0, 3, 5, 7, 10, and 15 days by using GC-MS analysis. The DBP standard exhibited sharp peaks at a retention time of 17.54 min (Figure 3a). A calibration curve was prepared with the tested concentrations (100- 0.01 µg mL^−1^). Confirmation of DBP was completed using its molecular ion peak at *m/z* 278 (Figure 3b), retention index and mass fragmentation pattern, which showed daughter ion peaks at *m*/*z* 223 and 205 after the sequential loss of butyl (-C_4_H_9_) and hydroxy (-OH) moieties, respectively. Additionally, other daughter ion peaks were observed at *m/z* 149 and 104, originating due to the subsequent removal of butyl (-C_4_H_9_) and acidic (-COOH) moieties, respectively (Figure 3c). The corresponding limit of detection, quantification, and recovery % of DBP was calculated to be 0.03, 0.5 mg mL^−1^ and 98.32%, respectively.

**Figure 4:**
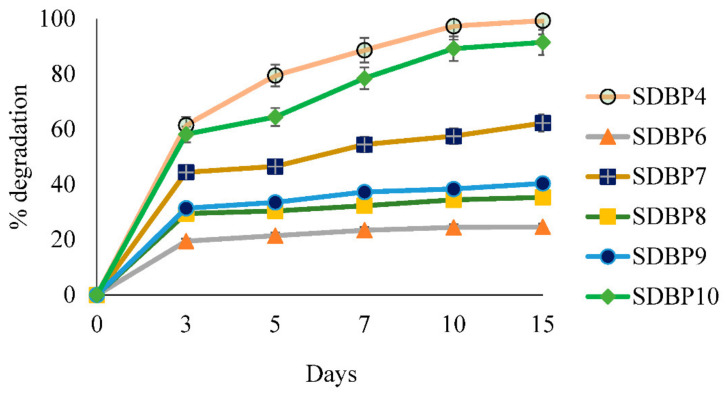
Extent of degradation of DBP by various isolates over the period.

The disappearance percentage of DBP is shown in Figure 4. Though the control without inoculation showed no degradation throughout incubation, adding fungi had a contrasting effect. It was evident that the initial DBP concentration (*C_i_*) of 98.28 mg L^1^ continued declining in the presence of all isolates concerning time, except for SDBP6 and SDBP8, which did not reduce significantly between the 10th and 15th day. Though SDBP7 degraded 44.41% on the 3rd day, at the end of the 15th day, it could only metabolize up to 62.27%, showing its inability to use DBP at later stages of growth. In contrast, SDBP4 mineralized 61.49, 79.48, 88.65 and 97.38% DBP on the 3rd, 5th, 7th and 10th days, respectively.

### 3.4. Molecular Identification of SDBP4

**Figure 5:**
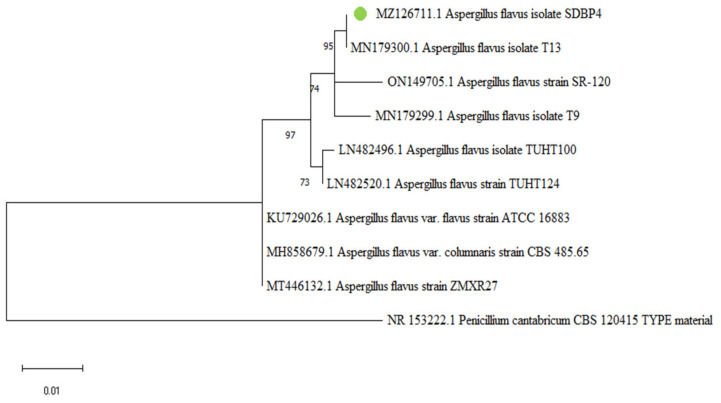
Phylogenetic tree of *Aspergillus flavus* SDBP4 using the maximum likelihood method. Bootstrap values (%) are denoted at the nodes.

The isolated strain SDBP4 was identified as *Aspergillus flavus* using NCBI BLAST_N_ of the ITS region of ribosomal DNA. Furthermore, sequence alignment revealed that SDBP4 is phylogenetically related to A. flavus T13 (Accession no. MN179300) with a similarity index of 99.29% (Figure 5). The sequence was deposited in the GenBank database under accession no. MZ126711.

### 3.5. Metabolites and Proposed Pathway

**Figure 6:**
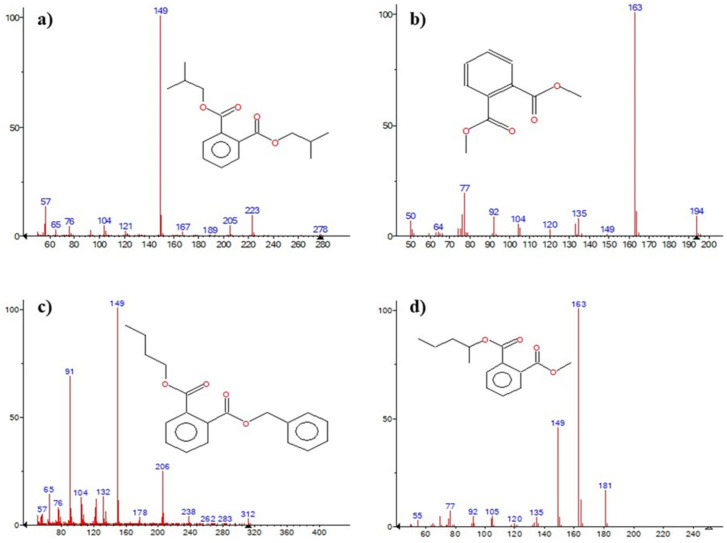
Mass fragmentation patterns of (**a**) Di-iso-butyl phthalate, (**b**) Dimethyl-phthalate, (**c**) Benzyl butyl phthalate and (**d**) Phthalic acid identified using GC-MS during DBP degradation by SDBP4.

The biodegradation of DBP at different time points generated useful information regarding degraded metabolites as intermediates. The GC-MS total ion chromatogram (TIC) at different time intervals displayed various peaks corresponding to DBP metabolites such as di-iso-butyl-phthalate (DIBP), dimethyl-phthalate (DMP), benzyl-butyl-phthalate (BBP), phthalic acid (PA). Further, the metabolites were characterized using their retention index, library matching and respective mass fragmentation pattern (Figure 6). After the 3rd day of inoculation, the samples exhibited another sharp peak at R_t_ 15.79 min along with the most abundant DBP (*m/z* 278). The additional compound was identified as DIBP (*m/z* 278), consisting of fragment ion peaks at *m/z* 223, 205, 167, 160, 132, 104 and 93. Interestingly, mono-butyl-phthalate (MBP) was not detected on the 3rd day in the inoculated samples. It was speculated that the conversion of DBP to MBP was faster (before 72 h of incubation) employing esterase and, therefore, could not be detected. After the 5th day of inoculation, the treated samples showed two major peaks at R_t_ 7.78 and 25.53 min, corresponding to DMP (*m/z* 194) and BBP (*m/z* 312). Table 2 displays the major fragment ion peaks. DMP exhibited fragment ion peaks at *m/z* 194, 149, 165, 104 and 77, while BBP exhibited fragment ion peaks at *m/z* 295, 238, 206, 178, 123, 91 and 65. The treated samples, after the 5th and 7th days of inoculation, exhibited a characteristic molecular ion peak at R_t_ 12.33 min of PA (*m/z* 166), which was consistent over the period. The major fragment ion peaks of PA were identified at *m/z* 181, 149, 135, 92 and 77.

**Figure 7:**
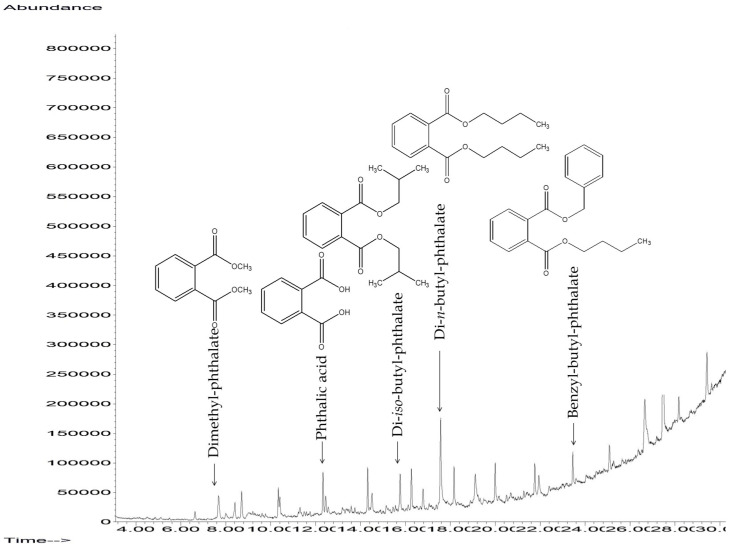
Characteristic peaks of different intermediates identified using GC-MS during the degradation of parent molecule DBP by SDBP4. The structures were constructed using ChemDraw JS 19.0.0.

**Figure 8:**
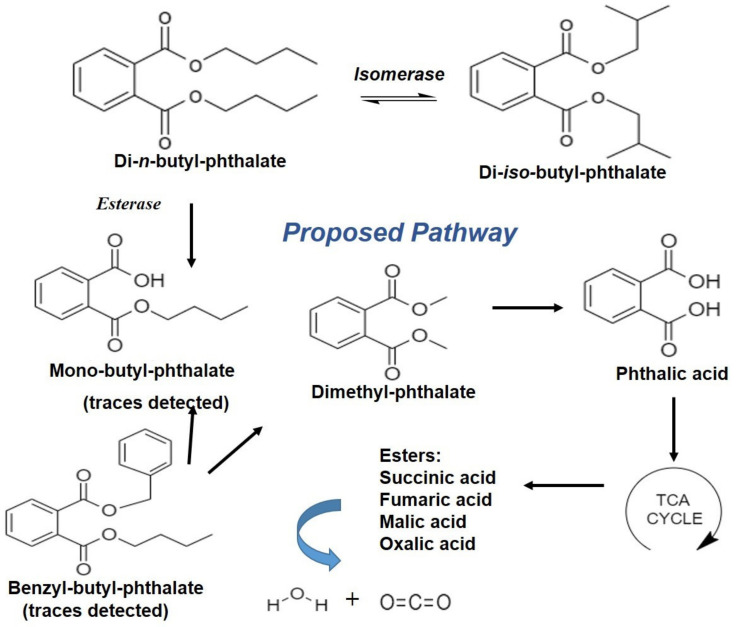
Proposed metabolic pathway of DBP degradation by SDBP. The structures were constructed using ChemDraw JS 19.0.0.

Figure 7 presents the metabolic intermediates identified in the degradation process of DBP after the 7th day of inoculation. Notwithstanding, long-chain hydrocarbons such as nonadecane, octadecane, heptadecane, hexadecane, pentadecane, tetradecane, undecane, eicosane, heneicosane and docosane along with long-chain fatty acids such as octadecanoic acid and decanedioic acid were identified using GC-MS chromatograms. It was evident from the GC-MS spectra that the relative content of the metabolites decreased over time and none of the intermediate phthalates were detected after the 15th day in the inoculated samples. Along with the degradation process, the relative peak area of DBP disappeared with the generation of metabolites. Intermediate metabolite profiling of DBP by the strain SDBP4 demonstrated fast hydrolysis to transform into MBP and other metabolites, DMP and BBP. BBP formation could result from the cyclization of the intermediate to form a compound with a closed phenyl ring. Further, the major metabolite, PA, was produced by hydrolysis through the de-esterification pathway (Figure 8).

## 4. Discussion

Phthalate esters are one of the toxic and harmful compounds that can accumulate in the environment in different ways. In general, various processes such as adsorption, absorption, photochemical oxidation and ionic interactions have been used for degradation, but none of these methods are efficient [2]. Microbial-based degradation is one of the most promising methods currently used for the degradation of various toxic or hazardous chemical compounds. Although, the consumption of these xenobiotic compounds for cellular carbon largely depends on their structural complexity as well as the efficiency of the microbial strain. Studies suggest that microorganisms easily consume short-chain phthalates (such as DBP) as compared to long-chain ones (such as DEHP) [18]. Some microorganisms including fungi have shown excellent and rapid degradation capacities of phthalate esters, particularly DBP [26,39,40,48]. However, the present fungi have been indigenous to toxic environments, such as sanitary landfill soil, that are rich in organic pollutants and nutrients such as N, P, K, copper, iron, manganese and zinc (unpublished data). For the first time, the present study highlights the highest DBP degradation by *A. flavus,* which is indigenous to the sanitary landfill.

A DBP-supplemented medium was inoculated with six fungi, viz. SDBP4, SDBP6, SDBP7, SDBP8, SDBP9 and SDBP10 individually, and growth parameters such as biomass, total soluble proteins and esterase production were assessed during different time intervals (Figure 2a,b). During degradation, the biomass and total soluble protein content in the medium of different isolates increased as time progressed. SDBP4 showed an increasing trend in biomass production and total protein content during DBP degradation and outperformed the other isolates. A similar trend in plasticizer degradation has been reported, which showed variable growth patterns by different microorganisms. This variation is due to the differences in substrate utilizing the capacity of microbial strains, concentration of substrate provided and time of incubation for degradation [31,40,49,50]. DBP metabolism also depends on enzymatic systems involving enzymes, such as esterases, and oxidoreductases, such as laccase, that possess hydrolytic activity [51,52]. However, the specific role of laccase in DBP degradation needs to be clarified [51]. Furthermore, a study confirmed a direct relationship of esterase in the mineralization of DBP by *Fusarium* sp. [42]. The present study also highlighted diversity in terms of esterase production potential among the isolated fungal strains, particularly by *A. flavus* (identified in a further section) for the first time.

As evident in Figure 4, DBP metabolism by fungi in the current investigation was positively correlated with time. The result was in accordance with another study performed with *F. culmorum* and *F. oxysporum* that exhibited an increase in degradation of DBP over time, although with different potentials [42]. On the 15th day, SDBP4 degraded 99.34% of the DBP present in the medium, while SDBP6 could degrade only 29.44%, which showed distinct variability in DBP assimilation among the isolates. A study including different fungi: *Ascocoryne* sp., *Phoma* sp., *Clavariopsis aquatica* and *Paradendryphiella arenariae* also exhibited differences in the consumption of DBP, which supports our results [41]. Thus, SDBP4 was further identified at the molecular level due to its highest degradation capacity. Further, degradation rate kinetics could be fitted to the first-order kinetic equation, *k* = 1/*t* (*lnC*_0_/*C_t_*), where *C*_0_ is the apparent initial concentration of DBP (µg/mL), *C_t_* is the concentration after a lapse of time *t* (days), and *k* is the degradation rate constant. The half-life (*t*_1/2_) value may be calculated from the respective *k* value [53].

Sporadic reports have been found about various metabolites of DBP degradation caused by potential fungi [38,39,40]. In addition, the intermediate metabolites were also found to be characterized based on the GC-MS total ion chromatogram in a previous study [54]. The DBP degradation pathway often involves two significant steps: (i) conversion of DBP to PA and (ii) PA to carbon dioxide [55]. Another study demonstrated biphasic degradation of DBP with the formation of monomeric ester, MBP, mediated by esterase and finally conversion to PA [56]. Interestingly, in the present study, MBP was not found throughout degradation, which has been reported to be of similar or higher toxicity than DBP [57,58].

Furthermore, the conversion of PA to protocatechuate under an aerobic environment has also been suggested by a few researchers, who reported the appearance of pyruvate as the end product [59]. The occurrence of diethyl phthalate (DEP) and DMP as prominent intermediates in the β-oxidation of DBP mediated by *Rhizobium* sp. LMB-1 has been proposed. Additionally, the immediate hydrolysis of DEP to produce tartaric acid with the generation of PA as an intermediate was further suggested [35]. A similar DEP formation phenomenon has been documented during the degradation of DBP [29]. Contrastingly, DEP did not appear in our study.

Nevertheless, DMP was identified as one of the primary metabolites. DBP is metabolized to produce DMP and MBP (traces) by dealkylation, which was converted to the most abundant PA. In a similar line of study, DMP esters were degraded within 22 days with the inoculation of *A. versicolor* IRM4, which produced various intermediates such as monomethyl phthalate, monomethyl isophthalate, monomethyl terephthalate, PA, isophthalic and terephthalic acid [46]. Likewise, the biodegradation of DBP has been reported, indicating the pathway to produce MBP, and then to form PA by *Gordonia* sp. strain QH-11 and *Providencia* sp. 2D [33,60] corroborates with our study’s salient findings involving *A. flavus* SDBP4.

Interestingly, BBP was characterized as an intermediate in the degradation pathway of DBP, which was further transformed to DMP, and the process was further catalyzed by cutinase or esterase. In this context, a similar conversion process has been proposed using *Fusarium oxysporum* f. sp. *pisi* [61]. In fact, DBP-degraded metabolites were gradually utilized by the fungal strain SDBP4 as carbon sources, hence, they were not detected at the end of our study (15th day of inoculation). Furthermore, simple esters of malate, fumarate, oxalate and succinate were detected in traces only after the 15th day of incubation, suggesting the fungus’s simultaneous utilization of intermediate metabolites. Simultaneous conversion of di-*n*-octyl phthalate to PA by *Gordonia* sp. strain JDC-2 and PA to CO_2_ by *Arthrobacter* sp. strain JDC-32 has been reported in the literature [62].

## 5. Conclusions

The present study demonstrates the DBP degradation capacity of *A. flavus* SDBP4, isolated from a toxic sanitary landfill site. The isolate SDBP4 had the highest growth attributes (biomass and total soluble protein) in a DBP-amended medium, demonstrating the potential to utilize the pollutant as a source. Furthermore, it has the capacity to degrade the contaminant by employing esterases. A comprehensive biodegradation pathway of DBP has been proposed for the first time, which illustrates the detailed characterization of DIBP and BBP along with other commonly reported metabolites such as DMP and PA. The strain SDBP4 can have promising applications in the biodegradation of DBP in contaminated agricultural soils, landfills and other matrices, serving as an environment-friendly alternative. The study also suggests that landfill soils can harbor potent microorganisms, especially fungi, that can be suitable substitutes for bacteria in the bioremediation of harmful plasticizers such as DBP.

## Figures and Tables

**Table 1:** Morphological characteristic of DBP-degrading fungal isolates on potato dextrose agar.

Isolate	Colony Characteristics	Sporulation	Spore Colour
SDBP4	Raised, grainy, filiform	Yes	Light green
SDBP6	Circular, raised, entire	No	-
SDBP7	Irregular, flat, filiform	Yes	Dark green
SDBP8	Irregular, grainy, filiform	Yes	Dull green
SDBP9	Raised, Irregular, filiform	Yes	Bright dark green
SDBP10	Raised, powdery, filiform	Yes	Dull green

**Table 2:** GC-MS identification of DBP and its intermediates.

R_t_ (min)	Compound			Molecular Ion Peak (*m/z*)	Base Peak (*m/z*)	Fragment Ions
		RI^lit^	^next^			
7.78	DMP	1466	1465	194	163	194, 149, 135, 104, 77
12.33	PA	1836	1839	250	163	181, 149, 135, 92, 77
15.79	DIP	1871	1873	278	149	223, 205, 167, 160, 132, 104, 93
17.54	DBP	1967	1969	278	149	223, 205, 167, 160, 121, 104, 93
25.53	BBP	2356	2359	312	149	295, 238, 206, 178, 123, 91, 65

Note: RI^(lit)^: Retention index on HP-5MS column reported in the literature. RI^(exp)^: Retention index on HP-5MS column, experimentally determined using homologous series of C8–C30 alkanes. DBP: di-*n*-butyl phthalate, DIBP: di-iso-butyl phthalate, DMP: dimethyl phthalate, BBP: benzyl-butyl-phthalate, PA: phthalic acid. PA was estimated as methyl-2-pentyl-phthalate.

## Data Availability

Not applicable.
